# Where is the hard-to-reach population? Spatial analysis from a cross-sectional study on the access to bed net and malaria vaccine in the Lake Victoria Region, Kenya

**DOI:** 10.1186/s12936-025-05280-2

**Published:** 2025-02-12

**Authors:** Yura K. Ko, Wataru Kagaya, Daisuke Yoneoka, James Kongere, Victor Opiyo, Jared Oginga, Protus Omondi, Kelvin B. Musyoka, Chim W. Chan, Bernard N. Kanoi, Jesse Gitaka, Akira Kaneko

**Affiliations:** 1 Department of Microbiology, Tumor and Cell Biology (MTC), Karolinska Institutet, Solna, Sweden; 2 Department of Virology, Tohoku University Graduate School of Medicine, Sendai, Japan; 3 Department of Ecoepidemiology, Institute of Tropical Medicine (NEKKEN), Nagasaki University, Nagasaki, Japan; 4 Center for Surveillance, Immunization, and Epidemiologic Research, National Institute of Infectious Diseases, Tokyo, Japan; 5 Department of Virology and Parasitology, Graduate School of Medicine, Osaka Metropolitan University, Osaka, Japan; 6 Center for Research in Tropical Medicine and Community Development, Nairobi, Kenya; 7 Center for Research in Infectious Diseases, Directorate of Research and Innovation, Mount Kenya University, Thika, Kenya; 8 Center for Malaria Elimination, Mount Kenya University, Thika, Kenya; 9 Department of Vector Ecology and Environment, Institute of Tropical Medicine (NEKKEN), Nagasaki University, Nagasaki, Japan

**Keywords:** Malaria vaccine, LLIN, Vaccine uptake, Mass net distribution, RTS,S, Kenya, Spatial analysis, Access

## Abstract

**Background:**

Long-lasting insecticidal nets (LLIN) and vaccines are effective malaria control tools. However, inadequate uptake has been reported in countries where both interventions are available. To maximize the impact these tools provide, it is crucial to identify populations that are not being reached and the barriers to uptake.

**Methods:**

In a cross-sectional study conducted in April 2024 in Kanyamwa Kologi Ward in Homa Bay County, Kenya, 4,662 households in 58 randomely selected villages were visited and interviewed. The proportions of households that (1) received at least one new LLIN within the previous five months (net distribution), (2) reported all children used LLIN (net usage), (3) reported at least one child had received one dose of the RTS,S vaccine (vaccine uptake), and (4) reported all children had received four doses of the vaccine (vaccine completion) were examined. Bayesian spatial autoregression analyses were used to estimate adjusted odds ratio (aOR) and its credible intervals (CrI) to identify the association between the household-level characteristics and the four outcomes.

**Results:**

The overall uptake proportions were 89.9% for net distribution, 84.4% for net usage, 88.2% for vaccine uptake, and 53.7% for vaccine completion. All four outcomes showed geographical heterogeneity with significant (p < 0.05) Moran's I. Households headed by adults of > 40 years had higher odds of having received a new LLIN (aOR = 2.02, 95% CrI 1.02–5.42), having one child who had received one vaccine dose (aOR = 1.83, 0.69–4.66), and having all children fully vaccinated (aOR = 2.36, 1.09–5.46), but lower odds of net usage by all children (aOR = 0.62, 0.40–0.96). Households with five or more children had higher odds of having received a new LLIN (aOR = 5.36, 2.24–27.0) but lower odds of net usage by all children (aOR = 0.24, 0.14–0.38) and having all children fully vaccinated (aOR = 0.20, 0.04–0.61). Distance to the nearest health centre was negatively associated with all outcomes. Household wealth was positively associated with all outcomes.

**Conclusion:**

Uptake of LLIN and malaria vaccine in Homa Bay County, Kenya varied by geography and household characteristics. These findings suggest that different sets of actions should be considered to improve the coverage and compliance of these interventions in different areas.

**Supplementary Information:**

The online version contains supplementary material available at 10.1186/s12936-025-05280-2.

## Background

The current global malaria situation is concerning. The progress in the reduction of malaria cases and deaths has stalled since 2015, with an estimated 249 million global cases and 610,000 deaths in 2022 [1]. Furthermore, *Plasmodium falciparum* partially resistant to artemisinin and its derivatives used in first-line treatment for malaria, was reported in sub-Saharan Africa (SSA) in 2021[2], and is becoming a global threat [3]. Ensuring the universal coverage of existing interventions in targeted populations is a pillar in the global strategy for malaria [4].

The distribution of long-lasting insecticidal nets (LLIN) is the most widespread intervention of malaria control and has contributed to the remarkable reduction of malaria cases and deaths since the early 2000s [5]. The most widely used insecticides in SSA countries were pyrethroid-based, but the emergence and spread of insecticide resistance in mosquito vectors has become problematic across the region. In response, the World Health Organization (WHO) issued a conditional recommendation in 2017 for the use of PBO-pyrethroid nets as a strategy to manage insecticide resistance [6]. Several cluster randomized trials (CRTs) [7, 8] showed that PBO-pyrethroid nets have greater epidemiological and entomological efficacy compared to pyrethroid-only LLINs in areas of high pyrethroid resistance.

Two safe and protective malaria vaccines, RTS,S/AS01 and R21/Matrix-M have been registered and licensed in several African countries over the last two years. In October 2021, the WHO recommended that children in moderate-to-high malaria transmission settings in SSA receive the RTS,S/AS01 vaccine. RTS,S/AS01 vaccine showed a 36.3% clinical malaria reduction among children aged 5–17 months in its phase 3 trial [9], and 13% malaria-related child mortality reduction after nearly four years of pilot administration in Kenya, Malawi, and Ghana [10]. More recently, in December 2023, the WHO prequalified the R21/Matrix-M vaccine [11]. In its phase 3 trial, the R21/Matrix-M vaccine showed a 72% clinical malaria reduction among children aged 5–36 months in Burkina Faso [12].

Despite the availability of these effective malaria public health measures, inadequate uptake, commonly set at < 80%, has been reported in many countries [13, 14]. To fully utilize these tools, it is crucial to identify populations that are not being reached and to gather more evidence on the barriers to uptake across various regions in SSA. Previous studies have shown the relationship between certain factors, such as socioeconomic status and health service factors, and the uptake of LLINs and vaccines [15–18]. However, these variables are often geographically correlated, making it better to explicitly consider the spatial distribution to avoid biased estimates of association [19]. Employing spatial analysis to identify specific factors associated with the uptake of LLINs and vaccines can contribute new evidence to this field of study.

Mass net distributions are conducted in Kenya every three years; the most recent campaign was officially launched in November 2023 [20]. In the campaign, PBO-pyrethroid nets were distributed free-of-charge to the community to target universal coverage. Meanwhile, the pilot RTS,S vaccination programmes were initiated in eight counties in the western region of Kenya in 2019 [21]. One of the counties is Homa Bay County, where a baseline survey was conducted in April 2024 for a cluster-randomized trial to evaluate the protective efficacy of a novel vector control tool, Olyset^®^ Plus ceiling nets [22]. Here, leveraging the available cross-sectional data, the study aimed to describe the uptake of mass net distribution and malaria vaccine implementation in the Lake Victoria region of Kenya in addition to investigating the characteristics of households that do not adopt these public health measures by using spatial regression analysis. By examining both LLIN and vaccine uptake simultaneously in a spatial context, this study provides a unique opportunity to identify common and divergent factors influencing these crucial malaria control interventions.

## Methods

### Study area

The study area is Kanyamwa Kologi Ward in Ndhiwa Sub County, Homa Bay County. The details of the area are described elsewhere [22]. Briefly, the ward has one level-four hospital and seven health centres with an approximate population of 33,000. Agriculture is the primary economic activity, with sugarcane as a main commercial crop. Residents also keep animals such as cattle, sheep, goats, and poultry [23]. The area generally experiences a long rainy season from March to June and a short one from October to December. In the study area, the latest mass net distribution was conducted in November and December 2023. In addition, RTS,S/AS01 vaccination was introduced to the area in 2019. Children aged 6 to < 12 months were eligible for the first dose. The second and third doses were to be administered at least one month apart, while the fourth dose was scheduled to be given as soon as possible after the child reached 24 months of age, with an upper age limit of 3 years [24].

### Study design and data collection

A cross-sectional malaria survey was conducted from April 8 to May 3, 2024, as a baseline survey for the cluster randomized trial to evaluate the efficacy of ceiling nets in Kanyamwa Kologi Ward [22]. Among 58 randomly selected villages, survey teams consisting of community health promoters (CHPs) and laboratory technicians visited all house structures and administered a questionnaire to the mother or the head of the household. The questionnaire included demographics of all household members including name, age, and sex, information about household structures, assets, bed net possession. Additionally, information on bed net usage on the night before the survey and malaria vaccination history was collected for children under 15 years old. It should be noted that the questionnaire was primarily designed for covariate randomization in the cluster-randomized trial rather than for this study. The questionnaire used in the 2020 Kenya Malaria Indicator Survey (KMIS) was modified. Questionnaire responses were collected by CHPs using the Research Electronic Data Capture (REDCap) software on electronic tablets [25].

### Descriptive analysis

Four binary variables at the household level were examined: (1) whether the household received at least one new PBO-pyrethroid net through the mass distribution campaign in 2023 (net distribution), (2) whether all children under 15 years old in the household used a bed net (regardless of whether it was a PBO-net or other LLINs) on the night before the survey (net usage), (3) whether at least one child aged 2–5 years in the household received a single dose of the malaria vaccine (vaccine uptake), (4) whether all children aged 2–5 years in the household received all four doses of the malaria vaccine (vaccine completion). The proportion of each variable was calculated using different denominators. For net distribution, all households with and without children were included; for net usage, only households with children under 15 years were included; for vaccine uptake and completion, only households with children aged 2–5 years were included. As explanatory variables, four categorical variables were targeted: (1) the mean age of the household head and its spouse (15–24, 25–40, and ≥ 41), (2) the number of children under 15 years old in the household (0, 1–2, 3–4, and ≥ 5), (3) distance to the nearest health centre (≤ 1 km, ≤ 2 km, and > 2 km), (4) the quantile of wealth index (low, middle, and high). The mean age of the household head and its spouse was selected based on the assumption that these individuals, including the spouse, collectively have the most influence within the household, rather than the household head alone. The wealth index was calculated according to the Demographic and Health Survey (DHS) guidelines [26]. To obtain a comprehensive overview, the four outcomes were summarized, stratified by health centres and a 500-m square grid, in addition to the explanatory variables.

### Statistical analysis

Spatial correlation in each of the four variables was assessed using Moran’s I. Then, a logistic regression model with a conditional autoregressive (CAR) Bayes regression framework was applied to each of the four binary outcomes by following equations:

$$\text{log}\left(\frac{{p}_{i}}{1-{p}_{i}}\right) = {{\varvec{x}}}_{i}^{T}{\varvec{\beta}}+{z}_{i}$$

where ${p}_{i}$ is the probability of the outcome of household $i$, ${{\varvec{x}}}_{i}$ is the covariate vector including (a) the mean age of the household head and its spouse, (b) the number of children per household, (c) distance to the nearest health centre, and (d) wealth index. These four variables were classified into three or four categories. ${z}_{i}$ is spatially structured random effects of the Leroux model, which assumes the (conditional) normal distribution *N*(), as follows:

$${z}_{i}|{z}_{i\ne j} \sim N(\frac{\rho \sum_{j}{w}_{ij}}{\rho \sum_{j}{w}_{ij}+1-\rho },\frac{{\tau }^{2}}{\rho \sum_{j}{w}_{ij}+1-\rho })$$

where ${w}_{ij}$ is the element of the spatial weights matrix, defined as 1 if the distance between ith and jth households is within 1 km and 0 otherwise. Parameters $({\varvec{\beta}}$, $\rho$,$\tau$) were estimated by using the Markov Chain Monte Carlo approach with four chains and 20,000 iterations including 500 burn-in periods by CARBayes package in R. The adjusted odds ration (aOR) of the probability of each outcome was estimated by $\text{exp}\left(\widehat{\beta }\right)$ with a 95% credible interval (95% CrI). Spatial autocorrelation is controlled by the parameters of ρ and τ. In addition, the model performance and fitting were evaluated by using the Deviance Information Criterion (DIC) and Widely Applicable Information Criterion (WAIC) for both types of CAR models and generalized linear models (GLMs) that do not incorporate a spatial weight matrix.

### Ethics declarations

Ethical approvals were obtained from the Mount Kenya University Intituitional Scientific Ethics Review Committee (MKU-ISERC) (approval number: 2565) and the Ethics Committee in Osaka Metropolitan University (approval number: 2024–068). Printed consent forms were read and explained to every house structure head/caregiver and their signatures captured electronically through electronic tablets during the survey.

## Results

### Uptake of the new LLINs and malaria vaccines

**Fig. 1:**
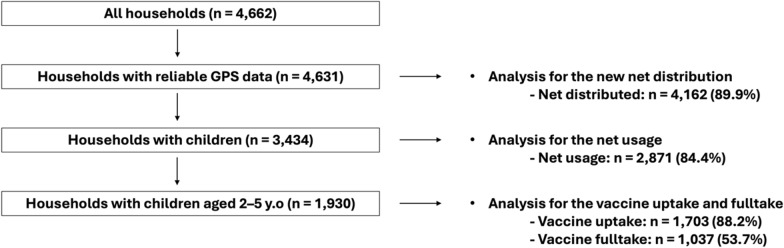
The number of households included in the study and for each analysis and the proportion of each outcome

Data were collected from 4,662 households in the study area; 31 were outside of Kanyamwa Kologi Ward and excluded. Among the remaining 4,631 households (80 households per village), 3,434 had at least one child under 15 years and 1,930 had at least one child aged 2–5 years. The overall uptake proportions for each outcome were 89.9% for net distribution, 84.4% for net usage, 88.2% for vaccine uptake, and 53.7% for full vaccination (Fig. 1).

**Fig. 2:**
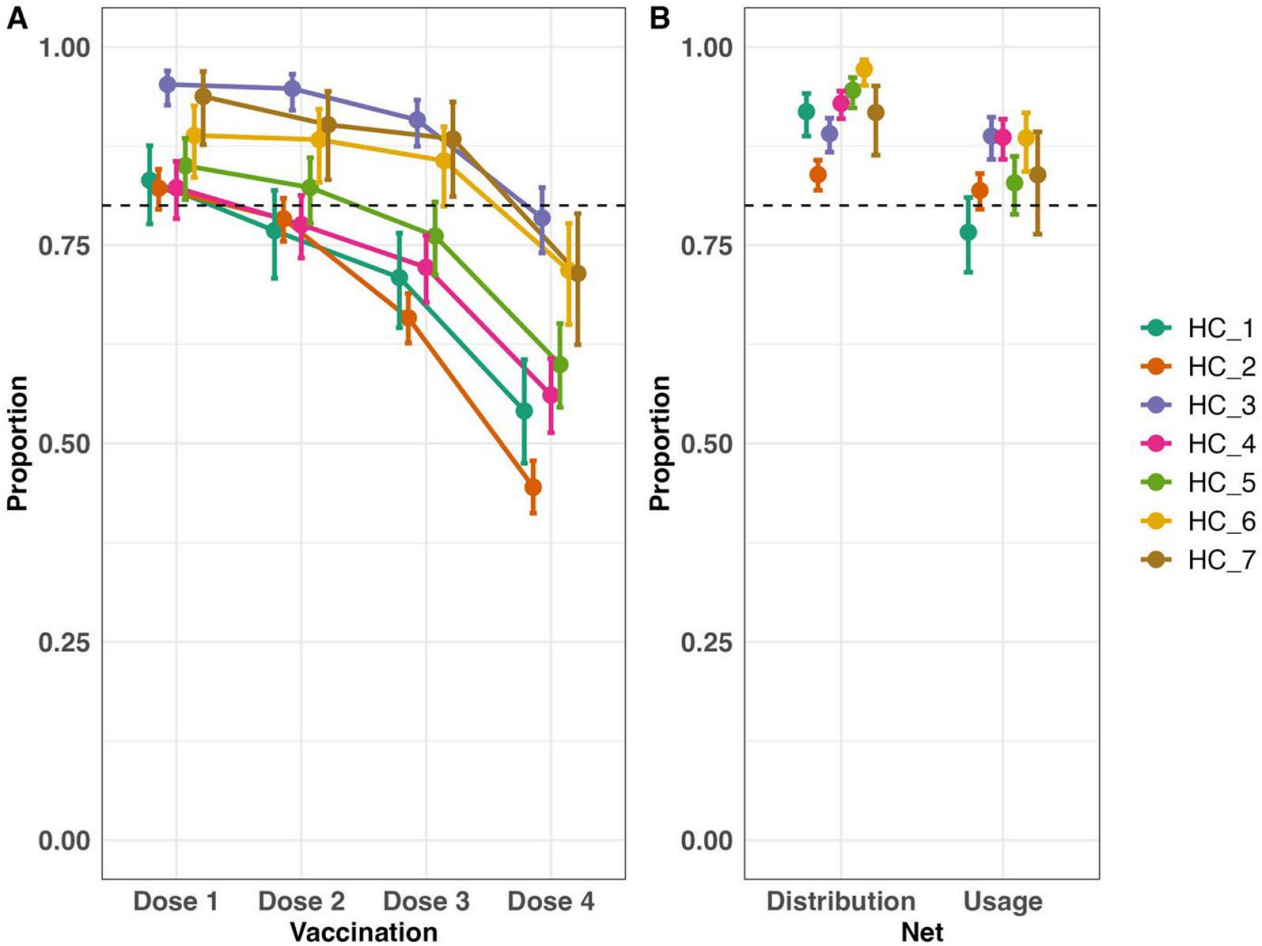
The uptake proportions for **A** easch dose of malaria vaccine and **B** net distribution and net usage by the responsible seven health centres. The error bars represent the 95% confidence intervals for each proportion

The uptake proportions for each dose of the malaria vaccine, net distribution, and net usage, stratified by the responsible seven health centres, are shown in Fig. 2 and Supplementary Table 1. All health centres achieved over 80% coverage for at least the first dose of the malaria vaccine. However, the coverage declined after dose 2, with only three health centres maintaining coverage above 80% for dose 3. By dose 4, none of the health centres had coverage above 80%; the uptake proportions varied from 44.5% to 78.4%. For net distribution, all health centres achieved 80% coverage, although net usage by children under 15 years old varied by area from 76.6% to 88.7% in the different health centres. Generally, vaccine coverage was associated with LLIN distribution and usage, though the association was not consistent across all health centres. For instance, HC_3, which had the highest vaccination coverage, also had the highest net usage but ranked second to last in LLIN distribution (Fig. 2).

**Fig. 3:**
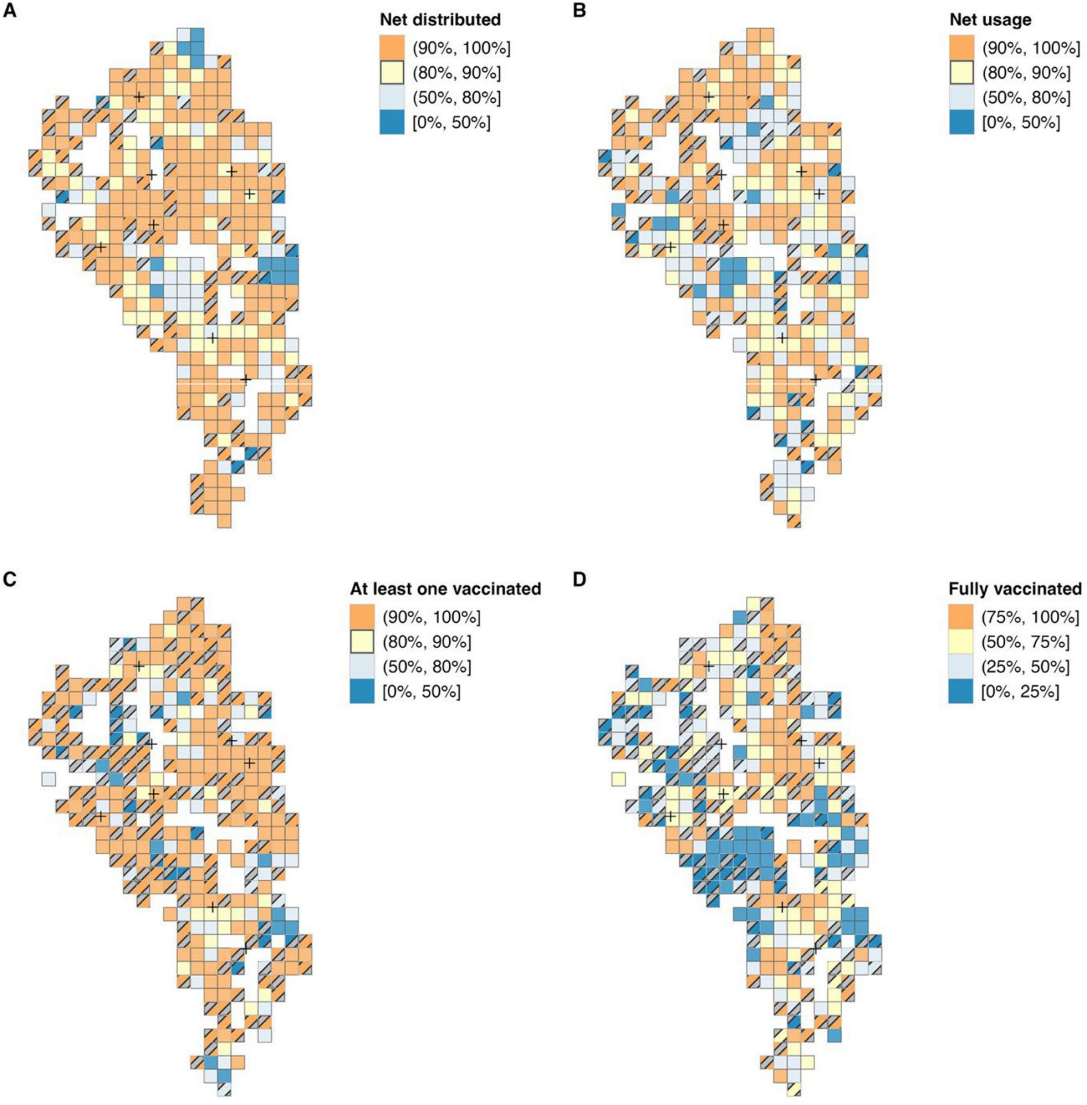
Spatial variation of **A** net distribution, **B** net usage, **C** vaccine uptake, and **D** vaccine completion in the study area. Stratified by its household-level proportion within a 500 m square grid area. Areas with a small number of households in the grid (fewer than 5) are indicated with diagonal lines. The crosses indicate health centres

The spatial distributions are shown in Fig. 3 and Supplementary Fig. 1, illustrating clear geographical aggregations for uptake of the LLINs and vaccines. Moran's I was calculated as 0.29 for net distribution, 0.08 for net usage, 0.19 for vaccine uptake, and 0.30 for vaccine completion, indicating strong evidence (P < 0.05) of a spatial correlation in all variables of interest. However, these geographical aggregations varied depending on each outcome. For instance, in the southernmost part of the study area, a higher proportion of households reported that none of their children had ever been vaccinated, despite the area's high coverage of net distribution.

### Factors associated with the uptake of the new LLINs and malaria vaccines

Table 1 shows aOR of each of four outcomes regarding (1) the mean age of the household head and its spouse, (2) the number of children per household, (3) distance to the nearest health centre, and (4) wealth index by the spatial regression. The household with an older mean age of the household head and its spouse had increased odds of net distribution, vaccine uptake, and vaccine completion. Compared to the household with the mean age categorized as 15–24, the aORs of net distribution, vaccine uptake, and vaccine completion in the household with the mean age over 40 years old were 2.02 (95% CrI 1.02–5.42), 1.83 (0.69–4.66), and 2.36 (1.09–5.46), respectively. For net usage, the aOR was 0.62 (0.40–0.96). If the household had more than two children, the odds of net usage and vaccine completion for all children were decreased, but more tend to get new LLIN. The aORs of net distribution, net usage, vaccine completion in the household with five or more children were 5.36 (2.24–27.0), 0.24 (0.14–0.38), and 0.20 (0.04–0.61), respectively. There was no clear trend about the association between the number of children and vaccine uptake. Distance to the nearest health centre was negatively associated with all four outcomes. Especially, if the household was located more than 2 km from the nearest health centre, the aOR of vaccine completion was the lowest at 0.12 (0.04–0.39). Finally, the wealth index of households was positively associated with all outcomes. Compared to the household with the lowest wealth index, the aORs of net distribution, net usage, vaccine uptake, and vaccine completion in the household with the highest wealth index were 2.74 (1.72–4.85), 1.81 (1.33–2.62), 1.95 (0.89–4.74), and 2.91 (1.32–7.41), respectively. The trend in net usage results did not change substantially when conditioning on households with at least one newly distributed LLIN (Supplementary Table 2). Supplementary Tables 3 and 4 showed the number and proportion of each stratified population regarding net and vaccine outcomes, respectively. With lower DIC and WAIC, CAR models for all four outcomes showed better fits to data than GLMs that did not incorporate a spatial weight matrix (Supplementary Table 5).

**Table 1:** Adjusted odds ratios (95% CrI) of the association between uptake of interventions and household-level attributes

	Net		Vaccine	
	Distribution	Usage	Uptake	Completion
Mean age of the household head and its spouse				
15–24	Ref	Ref	Ref	Ref
25–40	1.78 (0.93–4.78)	0.98 (0.64–1.53)	2.37 (1.09–7.53)	3.31 (1.13–9.81)
≥ 41	2.02 (1.02–5.42)	0.62 (0.40–0.96)	1.83 (0.69–4.66)	2.36 (1.09–5.46)
No. of children per HH				
0	Ref	–	–	–
1–2	1.35 (0.87–2.41)	Ref	Ref	Ref
3–4	2.03 (1.14–4.82)	0.43 (0.30–0.58)	0.83 (0.40–1.45)	0.43 (0.21–0.80)
≥ 5	5.36 (2.24–27.0)	0.24 (0.14–0.38)	1.30 (0.62–2.52)	0.20 (0.04–0.61)
Distance to the nearest health centre				
≤ 1 km	Ref	Ref	Ref	Ref
> 1 km to ≤ 2 km	0.91 (0.55–1.87)	0.62 (0.45–0.88)	0.74 (0.42–1.25)	0.38 (0.17–0.74)
> 2 km	0.42 (0.23–0.90)	0.66 (0.45–1.09)	0.84 (0.32–1.74)	0.12 (0.04–0.39)
Wealth index				
Low	Ref	Ref	Ref	Ref
Middle	2.06 (1.36–3.41)	1.36 (1.01–1.92)	1.43 (0.74–2.97)	1.00 (0.25–1.97)
High	2.74 (1.72–4.85)	1.81 (1.33–2.62)	1.95 (0.89–4.74)	2.91 (1.32–7.41)

## Discussion

This study described the uptakes of LLINs and the RTS,S/AS01 malaria vaccine in the Lake Victoria region of Kenya in 2024 and examined the factors influencing the uptakes. Although several reports from various areas have addressed LLIN [15, 27] and malaria vaccine [16, 17, 28] uptake independently, this is the first study to investigate simultaneously both interventions in the same area. Some common household-level characteristics were identified across the four outcomes: net distribution, net usage, vaccine uptake, and vaccine completion. Older average age of the household head and its spouse, proximity to a health centre, and higher household wealth were linked to a higher likelihood of receiving or complying with the interventions. However, geographical differences in adoption and compliance could be clearly discerned, and the spatial patterns differed among the measured outcomes. These findings suggest that different sets of actions should be considered to improve the coverage and compliance of these interventions in different areas.

Five months after the mass distribution, 89.9% of households were found to possess the distributed PBO-pyrethroid LLINs. Given that previous studies have indicated a rapid decline in net ownership beyond one year post-distribution [29, 30], this figure likely approximates the initial percentage of households that received the nets. This figure is slightly lower compared to the previous report in Uganda [15, 27], which showed 93.4% of households owned at least one new LLIN after 1–5 months of mass net distribution in 2020–2021. Moreover, Bhatt et al. estimated a median LLIN retention time of 23 months in 40 African countries [31]. Given the possibility of nets being damaged or lost over time, efforts should be made to get as close to 100% coverage of LLIN distribution as possible. The regression indicated that the households that did not receive the new LLINs were more likely to be headed by younger adults, have fewer children, live farther from a health centre, and be less wealthy. Given that nets can be an asset, the reasons for not receiving the new nets are likely to be different from the reasons for not using them or not having their children vaccinated. More likely, people wanted new nets but were omitted from the mass distribution. During the net distribution process, household heads were instructed to collect their new nets from designated locations such as health facilities and primary schools.

Households that did not receive new nets either failed to visit the collection point or were not included in the distribution registry. As these people who did not get nets could be socially isolated, it is important to incorporate these people into public policy targets. In addition, the mass net distribution campaign in 2023 was the first time an electronic registration system was used to identify eligible households in the study area. Anecdotally, some CHPs participating in both this study and the mass net distribution mentioned that there was some difficulty in utilizing the new technology, possibly resulting in the omission of some households. The Kenyan National Malaria Control Programme reported several challenges during the programme, including incomplete registration and distribution by some data collectors, difficulties in data access for CHPs, and issues related to mobile device fragmentation and usability [32].

Compared to net distribution, net usage showed less geographical clustering, suggesting that measures need to be directed towards a wider area population in order to increase net usage. Given that the regression results for net usage were similar regardless of conditioning on LLIN access (Supplementary Table 2), the findings on factors associated with net usage were not substantially influenced by net availability. A good approach to reaching a wider population is spreading the knowledge about malaria prevention. Kanyagarara et al*.* reported that knowledge of LLIN was associated with a 30–40% increased OR of net use in Zimbabwe and Zambia [33]. Additionally, previous studies in SSA reported that household size was strongly associated with inequality in the use of LLIN [27, 33, 34], which is consistent with the results of this study. Moreover, Tamari et al*.* reported sharing LLIN with two or more individuals may compromise its protective effect in areas close to the study site [35]. It is important to recognize that in large households, every member may not have equal access to LLINs, and a single net is often shared by several members.

Regarding malaria vaccine coverage, only the dose 1 uptake met the WHO coverage target of 80% across all health centres in the study area. After dose 2, coverage rates dropped dramatically, a trend commonly reported in other studies of malaria vaccines [14, 17, 28] and other multi-dose childhood vaccines [36, 37]. Since the regimen of the newly launched R21/Matrix-M vaccine is similar to that of the RTS,S vaccine, factors that lower the full uptake of the latter are likely to apply to the former. The regression analysis indicated that households that did not complete the full RTS,S regimen were typically headed by younger adults, had more children, lived farther from a health centre, and were less wealthy. One possible explanation for these findings is that households with these characteristics may not have the time and resources to ensure that all children are fully vaccinated. These populations should be prioritized to increase full-dose coverage of malaria vaccines. Although many efforts have been made to increase vaccination completion under the current immunization schedule, there have also been discussions about the possibility of changing the schedule itself. Alongside the development of a new vaccine with a shorter regimen [38], there is an ongoing study evaluating modification of the regimen and dosage of RTS,S vaccines without compromising their efficacy [39]. This should be focused on which regimen has a chance to increase the completion rate in each area setting based on the local context. This approach could be the ultimate form of tailor-made malaria control strategy [40].

Households with eligible children that did not take any dose of malaria vaccines differ from those with children who were not fully vaccinated based on the spatial distribution (Fig. 3) and the regression analysis (Table 1). Especially, proximity to the nearest health centre was not associated with vaccine uptake. This suggests that vaccine hesitancy may be more prominent in some areas and/or populations. Simbeye et al*.* reported that in Malawi, some did not take any dose of malaria vaccine because of religious beliefs [17]. In this study, some CHPs reported that certain religious leaders discouraged their followers from accessing publicly available health services, including malaria vaccination, as a reason for the abundance of malaria-unvaccinated households in their areas. In addition, it is known that trust in vaccines–not just malaria vaccines–has a profound effect on vaccine hesitancy in SSA countries [41, 42]. Unfried et al*.* showed that individuals’ trust in the government and society are key predictors of hesitancy towards polio, human papillomavirus, and COVID-19 vaccines in six countries (Ghana, Kenya, Nigeria, South Africa, Tanzania, and Uganda). Incorporating factors related to vaccine trust would provide a more holistic understanding of the barriers of malaria vaccine in future studies.

One of the strengths of this study is the incorporation of spatial aspects. Although there is growing awareness of spatial dependencies driven by proximity or shared social and environmental factors [43], there are still limited studies that explicitly account for spatial aspects in malaria research [44]. Ignoring spatial dependence in statistical models can lead to several issues, including biased estimates—such as failure to detect existing relationships and false identification of nonexistent relationships—and inflated significance levels due to underestimated standard errors [45]. While the regression analysis of this study reaffirmed the known factors influencing LLIN and vaccine uptake, it is noteworthy that similar associations were observed even after considering spatial correlation.

There are several limitations in this study. First, as baseline survey data from a cluster randomized trial that was not originally designed for this study were utilized, a sample size calculation was not conducted for the analysis. As a result, precise estimates may not have been achieved, particularly in the regression analysis. Nevertheless, each explanatory variable was categorized into three or four strata, which may suggest dose–response trends. Second, although CHPs were instructed to review the mother–child handbook to obtain malaria vaccination history, verbal reports were sometimes used if the mother had lost her handbook or if time constraints limited the survey. This introduces the possibility of recall bias, particularly regarding vaccination history. Third, information on the duration of residence for households at their current location was not collected. As a result, the analysis may include individuals who relocated from regions where the pilot vaccination programme was not implemented or who moved to the area after the mass net distribution. This may partially explain why younger household heads were less likely to possess new nets, as they are more likely to have established new households compared to older individuals. Fourth, data on the uptake of other vaccinations among the target population were not collected. Therefore, it remains unclear whether the findings are specific to the malaria vaccine or if other childhood vaccines face similar issues, such as lower coverage in the area.

In conclusion, as the characteristics of households who did not receive or comply with net distribution, net use, vaccination, and full vaccination overlap but differ slightly within the same area, tailored activities should be implemented to enhance the overall uptake of different interventions.

## Supplementary Information

Additional file 1

## Data Availability

The study regimes, consent forms, and study-related materials are accessible from the corresponding author. The corresponding author will make the de-identified datasets and source codes for all analysis available upon reasonable request.
