# Supplementary material for: Where is the hard-to-reach population? Spatial analysis from a cross-sectional study on the access to bed net and malaria vaccine in the Lake Victoria Region, Kenya
Source: Malar J. 2025 Feb 12;24:42. doi: 10.1186/s12936-025-05280-2 (PMC11823133; doi:10.1186/s12936-025-05280-2)
Supplement: Supplementary file 1 — Additional file 1 [file 12936_2025_5280_MOESM1_ESM.docx]

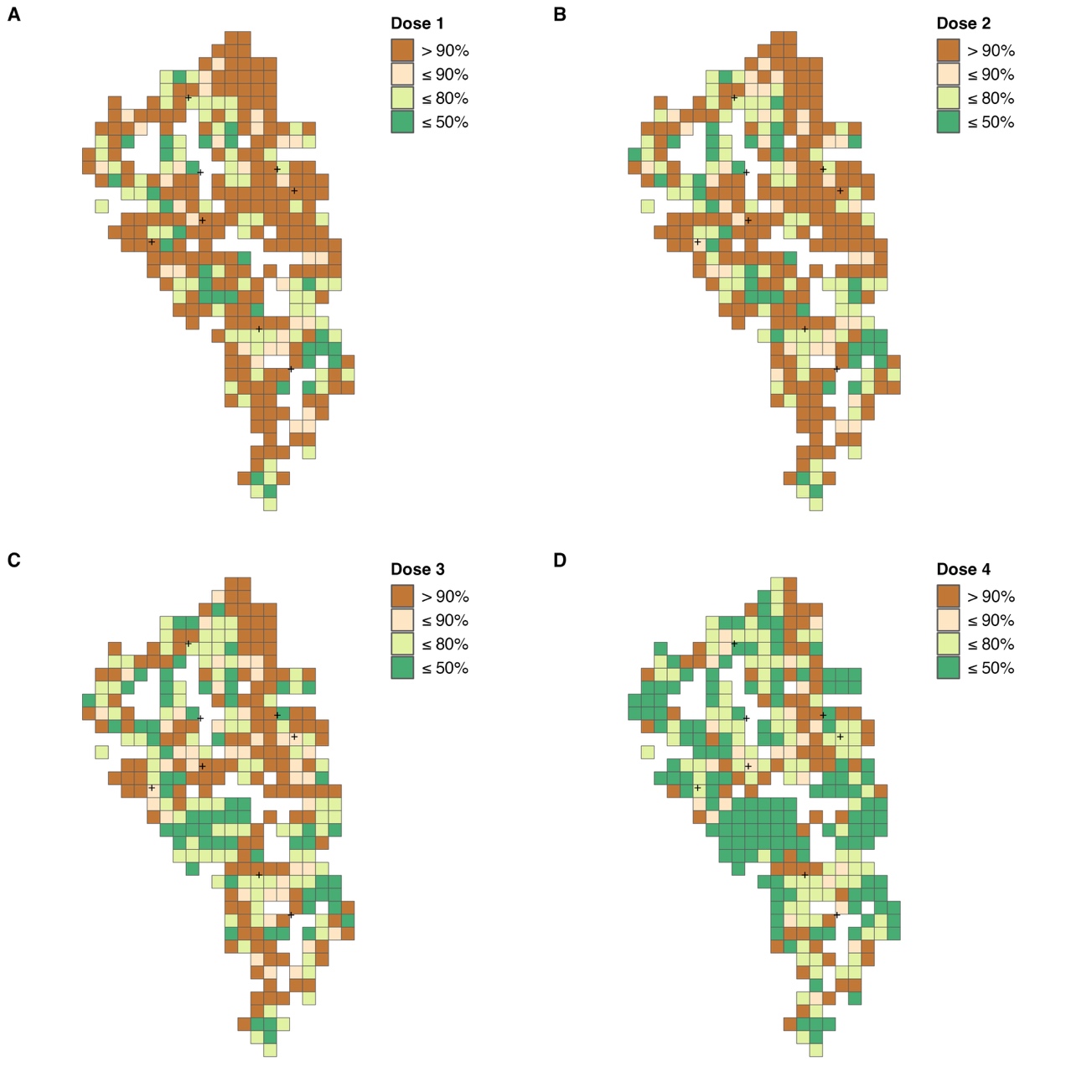


Supplementary Figure 1: Proportion of children aged 2–5 years with each dose of the malaria vaccine A) Dose 1, B) Dose 2, C) Dose 3, and D) Dose 4 in the study area. Stratified by its population-level proportion within a 500 m square grid area. The crosses indicate health centers.

Supplementary Table 1: The total number of children and households and uptake proportions for each dose of the malaria vaccine, net distribution, and net usage, stratified by the responsible seven health centers

|  | Vaccine | | | | | Net | | | |
| --- | --- | --- | --- | --- | --- | --- | --- | --- | --- |
|  | No. of children  aged 2–5 | Dose 1 | Dose 2 | Dose 3 | Dose 4 | No. of HHs | Net distibuted | No. of HHs  with children | Net usage |
| HC_1 | 220 | 83.2% | 76.8% | 70.9% | 54.1% | 404 | 91.8% | 308 | 76.6% |
| HC_2 | 881 | 82.2% | 78.3% | 65.8% | 44.5% | 1441 | 83.9% | 1126 | 81.9% |
| HC_3 | 380 | 95.3% | 94.7% | 90.8% | 78.4% | 813 | 89.1% | 542 | 88.7% |
| HC_4 | 428 | 82.2% | 77.6% | 72.2% | 56.1% | 842 | 92.9% | 605 | 88.6% |
| HC_5 | 327 | 85.0% | 82.3% | 76.1% | 59.9% | 548 | 94.5% | 408 | 82.8% |
| HC_6 | 188 | 88.8% | 88.3% | 85.6% | 71.8% | 426 | 97.2% | 287 | 88.5% |
| HC_7 | 112 | 93.8% | 90.2% | 88.4% | 71.4% | 157 | 91.7% | 124 | 83.9% |

HHs: Households, HC: Health center

Supplementary Table 2: Adjusted odds ratios (95% CrI) for the association between household LLIN usage and household-level attributes, conditioning on households with at least one newly distributed LLIN.

| Mean age of the household head and its spouse |  |
| --- | --- |
| 15–24 | ref |
| 25–34 | 1.27 (0.76-2.07) |
| 35­–44 | 0.73 (0.40-1.15) |
| No. of children per HH |  |
| 1–2 | ref |
| 3–4 | 0.38 (0.25-0.51) |
| 5– | 0.18 (0.10-0.29) |
| Distance to the nearest health center |  |
| ≤ 1km | ref |
| ≤ 2km | 0.59 (0.4-0.84) |
| > 2km | 0.60 (0.37-1.00) |
| Wealth index |  |
| Low | ref |
| Middle | 1.15 (0.84-1.59) |
| High | 1.45 (1.05-1.99) |

Supplementary Table 3: Characteristics of the households regarding distribution and usage of LLINs

|  | Net distributed or not^†^, N (%) | | Net used or not^‡^, N (%) | |
| --- | --- | --- | --- | --- |
|  | Distributed | Not distributed | All using | At least one not using |
| Mean age of the household head and its spouse |  |  |  |  |
| 15–24 | 214 (5.1) | 35 (7.5) | 160 (5.6) | 22 (4.2) |
| 25–40 | 1776 (42.7) | 197 (42.0) | 1477 (51.4) | 255 (48.2) |
| ≥41 | 1913 (46.0) | 200 (42.6) | 1079 (37.6) | 218 (41.2) |
| Unknown | 259 (6.2) | 37 (7.9) | 155 (5.4) | 34 (6.4) |
| No. of children per HH |  |  |  |  |
| 0 | 1058 (25.4) | 139 (29.6) | - | - |
| 1–2 | 1684 (40.5) | 209 (44.6) | 1650 (57.5) | 218 (41.2) |
| 3–4 | 1115 (26.8) | 107 (22.8) | 988 (34.4) | 229 (43.3) |
| ≥5 | 305 (7.3) | 14 (3.0) | 233 (8.1) | 82 (15.5) |
| Distance to the nearest health center |  |  |  |  |
| ≤ 1km | 1334 (32.1) | 86 (18.3) | 921 (32.1) | 112 (21.2) |
| >1km to ≤ 2km | 1737 (41.7) | 155 (33.0) | 1139 (39.7) | 237 (44.8) |
| > 2km | 1091 (26.2) | 228 (48.6) | 811 (28.2) | 180 (34.0) |
| Wealth index |  |  |  |  |
| Low | 1331 (32.0) | 213 (45.4) | 808 (28.1) | 164 (31) |
| Middle | 1397 (33.6) | 146 (31.1) | 975 (34.0) | 188 (35.5) |
| High | 1434 (34.5) | 110 (23.5) | 1088 (37.9) | 177 (33.5) |

^†^All households with and without children were included. ^‡^ Households with children under 15 years were included.

Supplementary Table 4: Characteristics of the households with children aged 2–5 years regarding malaria vaccination status

|  | Vaccinated or not, N (%) | | Fully vaccinated or not, N (%) | |
| --- | --- | --- | --- | --- |
|  | All not vaccinated | At least one child vaccinated | All fully vaccinated | At least one not fully vaccinated |
| Mean age of the household head and its spouse |  |  |  |  |
| 15–24 | 107 (6.3) | 21 (9.3) | 59 (5.7) | 69 (7.7) |
| 25–40 | 1035 (60.8) | 129 (56.8) | 638 (61.5) | 526 (58.9) |
| ≥41 | 481 (28.2) | 64 (28.2) | 295 (28.4) | 250 (28.0) |
| Unknown | 80 (4.7) | 13 (5.7) | 45 (4.3) | 48 (5.4) |
| No. of children per HH |  |  |  |  |
| 0–2 | 680 (39.9) | 97 (42.7) | 448 (43.2) | 329 (36.8) |
| 3–4 | 760 (44.6) | 104 (45.8) | 453 (43.7) | 411 (46.0) |
| ≥5 | 263 (15.4) | 26 (11.5) | 136 (13.1) | 153 (17.1) |
| Distance to the nearest health center |  |  |  |  |
| ≤ 1km | 525 (30.8) | 51 (22.5) | 385 (37.1) | 191 (21.4) |
| >1km to ≤ 2km | 689 (40.5) | 104 (45.8) | 405 (39.1) | 388 (43.4) |
| > 2km | 489 (28.7) | 72 (31.7) | 247 (23.8) | 314 (35.2) |
| Wealth index |  |  |  |  |
| Low | 480 (28.2) | 81 (35.7) | 290 (28.0) | 271 (30.3) |
| Middle | 575 (33.8) | 76 (33.5) | 323 (31.1) | 328 (36.7) |
| High | 648 (38.1) | 70 (30.8) | 424 (40.9) | 294 (32.9) |

Supplementary Table 5: DIC and WAIC of CAR models and GLMs that did not incorporate a spatial weight matrix.

|  | DIC | | WAIC | |
| --- | --- | --- | --- | --- |
|  | CAR | GLM | CAR | GLM |
| Net distribution | 2352.3 | 2901.0 | 2268.4 | 2901.4 |
| Net usage | 2711.0 | 2857.3 | 2676.0 | 2857.5 |
| Vaccine uptake | 1244.4 | 1400.7 | 1205.7 | 1401.0 |
| Vaccine completion | 1760.9 | 2590.6 | 1521.2 | 2590.7 |

DIC: Deviance Information Criterion

WAIC: Widely Applicable Information Criterion

CAR: conditional autoregressive

GLM: generalized linear model
